# The effect of local anesthetic on the hypersensitive and nonsensitive areas of the penis is different in primary premature ejaculation: a pilot study

**DOI:** 10.1093/sexmed/qfae020

**Published:** 2024-04-05

**Authors:** Lei Zheng, Le-Tian Wei, Song-Chunli, Wen-Rong Liu, Hui Jiang, Tao Jiang

**Affiliations:** Department of Andrology and Sexual Medicine, The Second Hospital of Dalian Medical University, Dalian 116023, China; Institution of Sexual Medicine, The Second Hospital of Dalian Medical University, Dalian 116023, China; Department of Andrology and Sexual Medicine, The Second Hospital of Dalian Medical University, Dalian 116023, China; Institution of Sexual Medicine, The Second Hospital of Dalian Medical University, Dalian 116023, China; Department of Neuroelectrophysiology, The First Hospital of Dalian Medical University, Dalian 116000, China; Department of Andrology and Sexual Medicine, The Second Hospital of Dalian Medical University, Dalian 116023, China; Institution of Sexual Medicine, The Second Hospital of Dalian Medical University, Dalian 116023, China; Department of Andrology, Peking University First Hospital, Beijing 100191, China; Department of Andrology and Sexual Medicine, The Second Hospital of Dalian Medical University, Dalian 116023, China; Institution of Sexual Medicine, The Second Hospital of Dalian Medical University, Dalian 116023, China

**Keywords:** sensitivity, penis, neurophysiological tests, local anesthetic

## Abstract

**Background:**

Penile hypersensitivity is not the whole penis, but rather only a part of the penis. Though local anesthetic can prolong intravaginal ejaculation latency time by reducing penile hypersensitivity, the effect on the hypersensitive and nonsensitive areas of penis is still unclear.

**Aim:**

The study aimed to explore whether the effect of local anesthetic on the hypersensitive and nonsensitive areas of the penis is different in premature ejaculation.

**Methods:**

Penile neurophysiological tests were performed on 290 patients with primary premature ejaculation. The sensory threshold, latency, and amplitude were recorded before and after the topical application of a local anesthetic (lidocaine cream) on the penis.

**Outcomes:**

Local anesthetics increased the sensory thresholds of hypersensitive and nonsensitive areas of the penis without difference but only prolonged the latency of the hypersensitive areas.

**Results:**

According to the neurophysiological results, 149 of 290 patients with primary premature ejaculation had normal penile sensitivity and 141 had penile hypersensitivity. While penile hypersensitivity does not necessarily mean that the whole penis is hypersensitive, and may be that only a part of the penis is hypersensitive, and we examined the following hypersensitivities: glans hypersensitivity only (14 cases), shaft hypersensitivity only (77 cases), and whole penis hypersensitivity (50 cases). Local anesthetics (lidocaine cream) increased the sensory thresholds of hypersensitive and nonsensitive areas of the penis without difference (*P* < .001) but only prolonged the latency of the hypersensitive areas (*P* < .001), and the latency of the nonsensitive areas was not different (*P* > .05).

**Clinical Implications:**

The present discovery implies that it is possible to improve ejaculation by applying local anesthetics externally to the hypersensitive areas of the penis to reduce the afferent local sensory signals, and improve intravaginal ejaculation latency time through accurately decreasing penile sensibility.

**Strengths & Limitations:**

This is the first large-sample study to explore the difference of local anesthetics’ effects on the hypersensitive and nonsensitive areas of the penis by means of neurophysiological methods in premature ejaculation. Our study exclusively examines alterations in penile evoked potential following electrical stimulation, which may not entirely encompass shifts in penile receptivity during sexual activity.

**Conclusion:**

The effects of local anesthetics on the same penis varied with penile sensitivity, and can only prolong the latency of hypersensitive area of the penis. The effect of local anesthetic on the hypersensitive and nonsensitive areas of the penis is different in premature ejaculation.

## Introduction

Intravaginal intercourse is the piston movement of the penis inside the vagina. The penis receives mechanical stimulation signals from sexual intercourse into electrical or chemical signals, then transmits these signals to the ejaculation-related central nervous system and produces a series of ejaculation-related responses until reaching the ejaculation threshold.^1^^,^^2^ In this process, the acceptance, transformation, and transmission of the signals are very important.

The penis is an important receptor in sexual intercourse: it transforms the information from sexual intercourse into a nerve impulse, transmits it to the central nervous system, and the central nervous system can analysis and integrate these signals. When these signals reach the ejaculation trigger point, the information will transmit to the effector, then ejaculate.^3^

Penile hypersensitivity is an important cause of premature ejaculation (PE).^4^^,^^5^Local anesthetics have been recognized for the treatment of PE by correcting penile hypersensitivity and reducing afferent local sensory signals.^6^ Wyllie et al^7^ found that local anesthetics on the glans could prolong the intravaginal ejaculation latency time by decreasing penile hypersensitivity. However, patients often complain of penile numbness, erectile dysfunction (ED), and diminished orgasmic pleasure.^7^ According to our previous research findings, differences in histology, nerve distribution, and receptors between the shaft and the glans of the penis produced a difference in sensitivity between the shaft and the glans in same penis. Penile hypersensitivity does not necessarily mean that the whole penis is hypersensitive, and may be that only a part of the penis is hypersensitive. We classify penile hypersensitivity into 3 categories, namely, glans, shaft, and whole penis hypersensitivity.^8^ Is there a difference in the effect of hypersensitive and nonsensitive areas after topical local anesthetic? We can reduce the sensitivity by accurately using local anesthetics only in hypersensitive areas, and on that basis reduce the amount of local anesthetics.

Taking the glans and shaft of the penis as the receptive area, the changes in the sensory threshold, latency, and amplitude of the hypersensitive and nonsensitive areas of the penis in each group before and after the application of the local anesthetics were detected by means of neurophysiological methods. To the best of our knowledge, this study is the first large-sample study to explore the difference of local anesthetics’ effects on the hypersensitive and nonsensitive areas of the penis by means of neurophysiological methods in PE.

## Methods

### Inclusion criteria

Data from 290 patients who attended the First Hospital of Dalian Medical University (Dalian, China) between July 2017 and March 2021 were collected. The inclusion criteria were as follows: (1) patients were over 24 years of age; (2) patients’ sexual partners were female and they had been engaging in regular intercourse for more than 6 months; (3) ejaculation often or always occurred before or within approximately 1 minute of vaginal penetration from the first sexual experience; (4) a Premature Ejaculation Diagnostic Tool score ≥11^9^; and (5) no surgery had been performed on the penis.

### Exclusion criteria

The following candidates were excluded: (1) patients with neurological disease or genital malformations, (2) patients with diseases of the endocrine system (diabetes, hyperthyroidism, etc.), (3) patients with ED, (4) patients with secondary PE, (5) patients with chronic prostatitis, (6) patients with mental health conditions, or (7) PE patients who had been taking selective serotonin reuptake inhibitors for a long period (>2 months).

### Examination method

Based on previous literature, patients with glans penis somatosensory evoked potential (GPSEP) latency below 40.83 ms or dorsal nerve somatosensory evoked potential (DNSEP) latency below 39.03 ms were defined as experiencing penile hypersensitivity.^10^ In this study, 290 patients with primary PE were divided into 4 groups according to their penile hypersensitivity area: glans hypersensitivity, shaft hypersensitivity, whole penis hypersensitivity, and whole penis nonsensitivity. This study was approved by the Ethics Review Board of the First Hospital of Dalian Medical University (Approval No. PJ-KS-KY-2022-361), and oral informed consent was obtained from all patients.

Neuroelectrophysiological testing methods were the following: the patient was instructed to be in a quiet state, excluding any interference; lighting and temperature were kept at a comfortable level; the patients were first informed of the main examination procedures and the precautions of the examination so that they could fully cooperate; each patient was asked to remain in the supine position and to expose the external genitalia and right forearm; and the surface of the patient’s glans penis and shaft was washed with saline. Electrophysiologic data were recorded using an electromyography/evoked potential apparatus (Synergy 10-conductorSynergy10-conductor, Oxford, England). The positive and negative electrodes of the ring electrodes were placed in the coronal groove of the penis and at the root of the penis, respectively. The grounding electrode was placed at the patient’s right wrist. Sheet electrodes were placed at the center of the subject’s head (Cz) and at the frontal parietal median (FPz) position by applying a conductive paste to reduce impedance, and tape was used to secure them. The recording and reference electrodes were correctly placed, and the impedance of the electrodes was made <5 KΩ. The participant was given a 5-minute acclimatization period before recording. The patient was instructed to relax, and the stimulation current was started at 0 mA and slowly increased. The threshold for DNSEPs was the intensity of the stimulation current at which the patient felt a slight pinprick sensation on the penile shaft. Thereafter, the current intensity was increased to 3 times this threshold and superimposed 200 times. The time required for the first evoked potential (EP) wave to appear was recorded as the DNSEP latency. The amplitude of the DNSEP at this time was recorded as the amplitude. GPSEPs were obtained by means of 2 sheet electrodes, which were placed on both sides of the penile urethral opening after being coated with a conductive paste, and the positions and settings of the grounding electrodes, scalp recording electrodes, and reference electrodes were the same as those of DNSEPs; they will not be described here. The threshold, latency, and amplitude of the neurophysiology recorded at this time were the data before the anesthetic. Next, lidocaine (10 g; Tongfang) was applied to the patient’s glans and shaft and spread evenly with a cotton swab. The patient was instructed to lie down for 10 minutes and then continue the previous operation, and the recorded data were the data after the anesthetic. According to the patients’ latency before the use of anesthetic, the patients were divided into a simple penis glans hypersensitivity group, a simple penis shaft hypersensitivity group, a whole penis hypersensitivity group, and a nonsensitive group, and the differences in the results before and after the anesthetic were compared.^11^

### Statistical methods

Data were statistically analyzed using SPSS 23.0 (IBM). First, the Kolmogorov-Smirnov test was used to test the normality of continuous variables. Variables that conformed to a normal distribution were expressed as mean ± SD. When variables were skewed, the median (interquartile range) was used. The latency and thresholds of the glans and shaft of the penis conformed to a normal distribution. The amplitudes of the glans and shaft did not conform to a normal distribution. Paired *t* tests were used for those that conformed to normal distribution, and Mann-Whitney *U* tests were used for those that did not conform to normal distribution. Unless otherwise noted, *P* < .05 was considered a statistically significant difference.

## Results

### General data

This study collected a total of 290 primary PE patients who attended the First Affiliated Hospital of Dalian Medical University during the period from July 2017 to March 2021, and collected general data such as age, height and weight, Premature Ejaculation Diagnostic Tool score, marital status, history of smoking, history of alcohol consumption, and neural electrodynamic data such as biosensory thresholds, latency, and waveforms of DNSEPs and GPSEPs. For characteristics of the 290 patients, see Table 1.

**Table 1:** Characteristics of the 290 patients.

Characteristic	Value
Age, y	29.04 ± 4.42
Height, cm	174.92 ± 4.52
Body mass, kg	71.21 ± 5.43
PEDT score	14.20 ± 2.14
Not married	202 (69.7)
Smoking	137 (47.2)
Alcohol consumption	228 (78.6)

Values are mean ± SD or n (%).

Abbreviation: PEDT, Premature Ejaculation Diagnostic Tool.

In this study, patients with PE were categorized into a simple glans penis hypersensitivity group (14 patients, 4.83%), a simple penile shaft hypersensitivity group (77 patients, 26.55%), a whole penis hypersensitivity group (50 patients, 17.24%), and nonsensitive group (149 patients, 51.38%) based on the latency. For details, see Table 2.

**Table 2:** Penile grouping of premature ejaculation patients.

GPSEP (ms)	DNSEP (ms)	Group	n
<40.83	≥39.03	Simple glans penis hypersensitivity	14
≥40.83	<39.03	Simple penile shaft hypersensitivity	77
<40.83	<39.03	Whole hypersensitivity	50
≥40.83	≥39.03	Nonsensitive	149

Abbreviations: DNSEP, dorsal nerve somatosensory evoked potential; GPSEP, glans penis somatosensory evoked potential.

### Differences in the simple glans hypersensitive group

**Figure 1:**
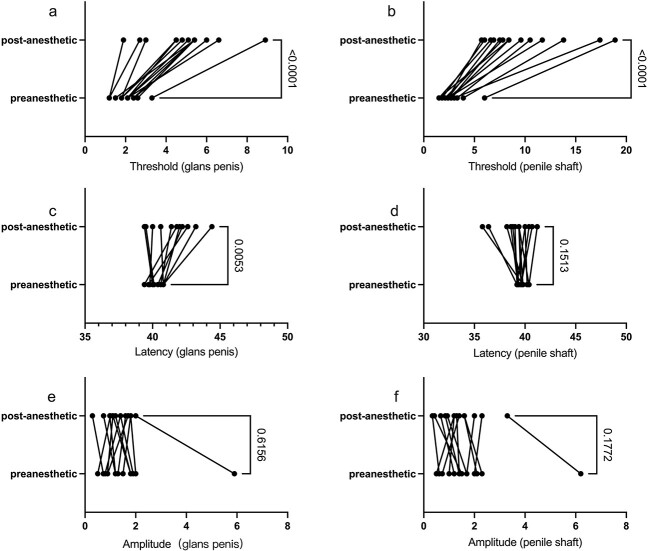
Differences in simple glans hypersensitive group. (A, B) The biosensory threshold of glans and shaft increased after the application of local anesthetics. (C, D) Only the latency of the glans was prolonged, not the shaft. (E, F). The amplitude did not change before and after the local anesthetic on the glans penis and the penile shaft.

In the simple glans hypersensitivity group, both biosensory thresholds of the glans and shaft were significantly increased (*P* < .001). However, only the latency of hypersensitive glans was significant prolonged (*P* = .0053) after the application of local anesthetics, and the latency of the nonsensitive shaft was not prolonged (*P* = .1513). Meanwhile, the amplitude did not change before and after the local anesthetics on the glans penis and the penile shaft, with *P* values of .6156 and .1772, respectively (Figure 1).

### Differences in the simple shaft hypersensitive group

**Figure 2:**
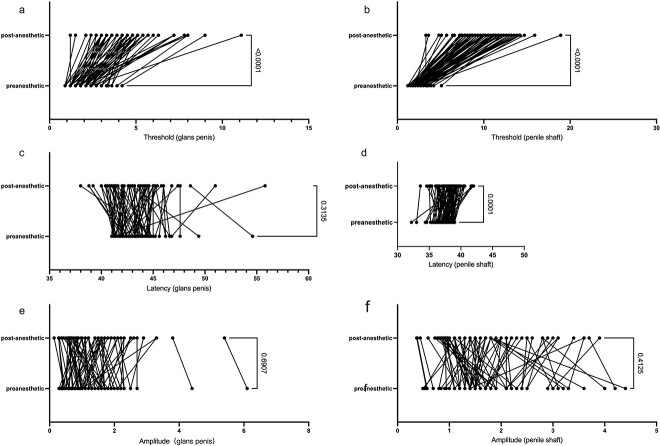
Differences in simple shaft hypersensitivity group. (A, B) The biosensory threshold of glans and shaft increased after the application of local anesthetics. (C, D) Only the latency of hypersensitive shaft was prolonged, not the glans. (E, F) The amplitude of the glans and shaft did not change before and after the local anesthetics.

In the simple shaft hypersensitivity group, the biosensory thresholds of the glans and shaft significantly increased after application of local anesthetics (*P* < .001), and only the latency of hypersensitive shaft was significantly prolonged (*P* < .001), while that of the glans was not prolonged (*P* = .3135). The amplitude of the glans penis and penile shaft did not change, with *P* values of .6907 and .4125, respectively (Figure 2).

### Differences in whole penis hypersensitive group

**Figure 3:**
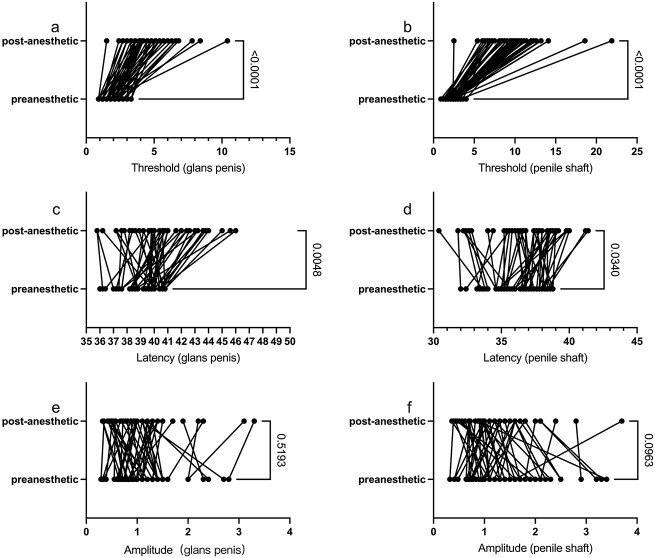
Differences in the whole penis hypersensitivity group. (A, B) The biosensory threshold of the glans and shaft increased after the application of local anesthetics. (C, D) The latency of the glans and shaft was prolonged. (E, F) The amplitude of the glans and shaft did not change before and after the local anesthetics.

In the whole penis hypersensitivity group, the biosensory thresholds of the glans and shaft significantly increased (*P* < .001), and the latency of the glans and shaft was prolonged after application of local anesthetics, with *P* values of .0048 and .0340, respectively, while the amplitude of the glans and shaft did not change, with *P* values of .5193 and .0963 (Figure 3).

### Differences in the nonsensitive group

**Figure 4:**
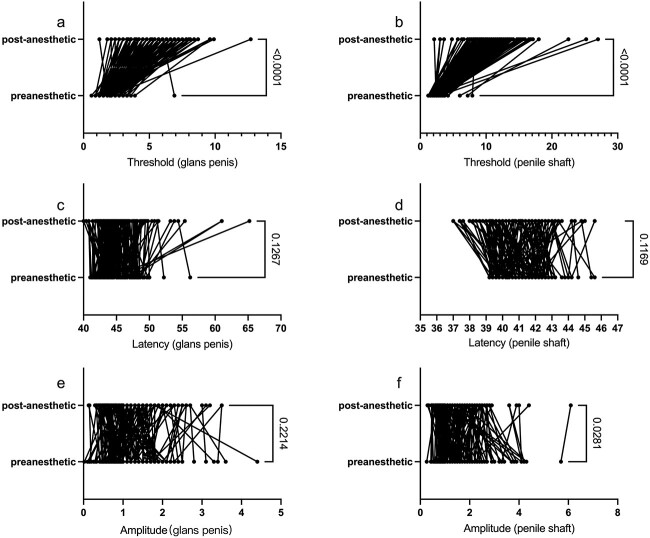
Differences in the nonsensitive group. (A, B) Both the biosensory threshold of the glans and shaft thresholds increased after the application of local anesthetics. (C, D) There was no change in the latency before and after the local anesthetics on the glans penis and the penile shaft. (E, F) The amplitude was elevated only in the shaft, not in the glans.

In the nonsensitive group, the biosensory thresholds of the glans and shaft in patients with PE significantly increased after the application of local anesthetics (*P* < .001), and the latency of the glans and shaft was not prolonged after application of local anesthetics, with *P* values of .1267 and .1169, respectively. At the same time, the amplitude of the shaft was elevated after the application of local anesthetics (*P* = .0281), and the amplitude of the glans did not change after the application of local anesthetics (*P* = .2214) (Figure 4).

## Discussion

Penile hypersensitivity is an important cause of PE. Yang et al^10^ divided PE into sympathetic central sensitivity PE, penile sensitivity PE, mixed PE, and neurophysiologically normal PE through neurophysiological testing, and adopted corresponding treatment for different types of PE (ie, for sympathetic central sensitivity PE, an oral selective serotonin reuptake inhibitor can be chosen; for penile sensitivity PE, topical local anesthetics can be used).

The penis consists of 3 cylinders, namely, 2 in the penile corpus cavernosa and 1 in the urethral corpus cavernosum; the glans originates from the urethral corpus cavernosum, and the shaft mostly originates from the penile corpus cavernosum.^12^ In a study conducted by Diallo et al,^13^ it was revealed through computer-assisted techniques on 5 adult autopsies that the proximal region of the penis exhibited the presence of both somatosensory nerve fiber–specific antibodies (indicated by positive immunostaining with a specific antibody against PMP22+ [peripheral myelin protein-22]) and visceral motor nerve fiber–specific antibodies (with positive immunostaining of nitric oxide synthase, tyrosine hydroxylase, and vesicular acetylcholine transporter). In contrast, the distal one-third of the penis displayed a higher density of somatosensory fibers (PMP22+). The nerve of the glans is not just a simple continuation of the nerve of the shaft.^14^^,^^15^ There are differences in the innervation, histology, and receptors of the two, which results in differences in sensitivity. Penile hypersensitivity is not the whole penis, but rather only a part of the penis; there are hypersensitive areas of the penis, and the hypersensitive area may be the shaft, glans, or the entire penis. According to the different penile hypersensitivity area, we divided hypersensitive penis into 3 subtypes: simple glans hypersensitive, simple shaft hypersensitive, and whole penis hypersensitive.^8^

While the application of local anesthetics to treat PE has been recognized,^6^ patients often complain of side effects such as penile numbness, impaired erection, and diminished orgasmic pleasure due to the application dose and the inability to be precise in the topical application site. Can the side effects of the local anesthetics and the previously mentioned complaints be reduced if the hypersensitive areas of the penis are desensitized in a precise manner? For this reason, this study was conducted to investigate the differences in the effects of local anesthetics on the sensibility of the hypersensitive and nonsensitive areas of the penis.

### The effect of local anesthetics on the threshold of each group

After the action of the local anesthetic, the threshold of penile hypersensitive and nonsensitive areas in each group increased significantly, suggesting that a local anesthetic can increase the sensory threshold of the whole penis (including the sensitive and nonsensitive areas) and reduce the excitability of the whole penis only.

### The effect of local anesthetics on the latency of each group

After the application of local anesthetics, in the simple glans hypersensitivity group, only the latency of the glans was significantly prolonged, while the latency of shaft was not prolonged; in the simple shaft hypersensitivity group, only the latency of shaft significantly was prolonged, while the latency of glans was not prolonged; in the whole penis hypersensitivity group, the latency of the shaft and the glans were prolonged; and in the whole penis nonsensitive group, the latency of the shaft and the glans was not prolonged. This is an interesting phenomenon out of our expectation: after the application of local anesthetics, the latency of the hypersensitive area was significantly different than that of the nonsensitive areas of the penis and the latency of the hypersensitive area significantly was prolonged, while the latency of the nonsensitive area did not change.

### The effect of local anesthetics on the amplitude of each group

After the application of a local anesthetic, regarding the amplitude of the hypersensitive group, neither the hypersensitive nor the nonsensitive area decreased, but only in whole penis nonsensitive group was the amplitude of the penile shaft significantly reduced, suggesting that there may be neural differences between the two areas.

### Local anesthetics can prolong only the latency of the hypersensitive area, not the nonsensitive area

That the local anesthetics only prolonged the latency of the hypersensitive area reaffirms our previous finding that the perceptibility of the glans and the shaft are different.^8^ We confirmed the perceptibility differences between the shaft and glans through neurophysiological testing. For the first time in the international community, we put forward the idea that there is a hypersensitive area in the hypersensitive penis, and the hypersensitive penis is divided into at least 3 subtypes, namely, simple glans hypersensitivity, simple shaft hypersensitivity, and whole penis hypersensitivity. We guessed that after the application of local anesthetics, the sensory threshold of hypersensitive and nonsensitive areas would increase and the latency would prolong, but the results of this study are surprising. Only the sensory threshold increased without difference, and the latency was not prolonged; only the latency of hypersensitive area was prolonged, and the latency of nonsensitive area was not prolonged. The findings supported and verified again our previous findings that the penis shaft and glans sensibility are different; there is a different sensibility in the glans and shaft. A hypersensitive penis does not mean that the entire penis is hypersensitive, but rather that there are hypersensitive areas of the penis.

### The potential mechanism for local anesthetics only prolongs the latency in hypersensitive areas

First, regarding the mechanism of application of local anesthetics, local anesthetics like lidocaine exert their effects by interacting with nerve cell membranes.^16^ They obstruct the exchange of sodium across the cell membrane, thus preventing the influx of sodium through ion channels.^17^ This action diminishes the excitability of the nerve membrane and suppresses the transmission of neural action potentials.

Second, regarding the potential mechanism of local anesthetics selectively prolonging the latency of hypersensitive areas, a lot of factors influencing the adaptation and functionality of penile receptors, such as the energy conversion process of penile receptors; synaptic transmission properties between receptor cells and sensory nerve fibers; the function of ion channel. The latency of evoked potentials is related to nerve conduction velocity,^18^ nerve thickness,^19^ and the synapses.^20^ Lidocaine, the local anesthetic, modulates the functional state of ion channels, preventing the influx of sodium through these channels and reducing the nerve membrane’s excitability.^21^ However, changes of the ion channel state by local anesthetics were the same; only the latency of the hypersensitive area, and not the nonsensitive area, was significantly prolonged, suggesting that the receptor cells and sensory nerve fibers of hypersensitive and nonsensitive areas were different. This discrepancy suggests that there may be a biological basis for a notable distinction between the hypersensitive and nonsensitive areas in synaptic transmission or receptor cells.

## Research significance

This study investigated alterations in sensitivity across various subtypes of both hypersensitive and nonsensitive areas before and after the application of a local anesthetic. Our findings indicated that local anesthetics could selectively only prolong the latency of hypersensitive areas, and not the nonsensitive area, while the sensory threshold of the hypersensitive areas and nonsensitive area was uniformly increased. The desensitization effect of the local anesthetic was different between the hypersensitive and nonsensitive areas in PE.

Although local anesthetics can effectively treat PE, patients frequently complain of discomfort such as penile numbness, unsatisfactory erection, and diminished orgasmic pleasure. Our research has unveiled a significant insight: these anesthetics exclusively, selectively prolong the latency of hypersensitive areas. This discovery implies that whether it is possible to improve ejaculation by applying local anesthetics externally to the hypersensitive areas of the penis to reduce the afferent local sensory signals and improve intravaginal ejaculation latency time through accurately decreasing penile sensibility and reducing the aforementioned complaints to achieve more precise treatment requires in-depth study.

## Limitations

The primary sensory receptors in male genitalia consist of myelinated fine free nerve endings. These free nerve ending receptors can detect mechanical stimuli (such as stretching, vibration, touch, and pressure), as well as temperature and pain, converting diverse sensory signals into excitatory responses. Nevertheless, our study exclusively examines alterations in penile evoked potential following electrical stimulation, which may not entirely encompass shifts in penile receptivity during sexual activity.

## Conclusion

Local anesthetics can increase the threshold of hypersensitive and nonsensitive areas of the penis, but only prolonged the latency of the hypersensitive areas, not the nonsensitive areas. The pronounced variance in latency between these areas affirmed that the nerves in the glans are not merely a direct extension of those in the penile shaft, indicating disparities in penile receptivity.

However, sensory separation between the shaft and the glans of the penis reinforces our proposed concept: hypersensitive areas within the penis. It suggests potential biological distinctions between hypersensitive and nonsensitive areas in terms of receptor synaptic transmission, receptive cells, and sensory nerve fibers. In future investigations concerning PE, further precision should be applied by segregating the penile shaft from the glans penis and distinguishing between hypersensitive and nonsensitive areas.

## Data Availability

The data that support the findings of this study are available from the corresponding authors upon reasonable request.
