# Correction to: The effect of local anesthetic on the hypersensitive and nonsensitive areas of the penis is different in primary premature ejaculation: a pilot study

**DOI:** 10.1093/sexmed/qfae037

**Published:** 2024-05-17

This is a correction to: Lei Zheng, Le-Tian Wei, Song-Chunli, Wen-Rong Liu, Hui Jiang, Tao Jiang, The effect of local anesthetic on the hypersensitive and nonsensitive areas of the penis is different in primary premature ejaculation: a pilot study, *Sexual Medicine*, Volume 12, Issue 2, April 2024, https://doi.org/10.1093/sexmed/qfae020

The following changes have been made to the originally published manuscript.

Dr Tao Jiang is now also marked as corresponding author.

In **Author contributions** section, the final sentence is emended to read: “These authors contributed equally to this work: L.Z., L.-T.W.” instead of: “These authors contributed equally to this work.”

The emendations have been made to the article.

